# Supplementary figures and images for: Influence of observer-dependency on left ventricular hypertrabeculation mass measurement and its relationship with left ventricular volume and ejection fraction – comparison between manual and semiautomatic CMR image analysis methods
Source: PLoS One. 2020 Mar 11;15(3):e0230134. doi: 10.1371/journal.pone.0230134 (PMC7065796; doi:10.1371/journal.pone.0230134)

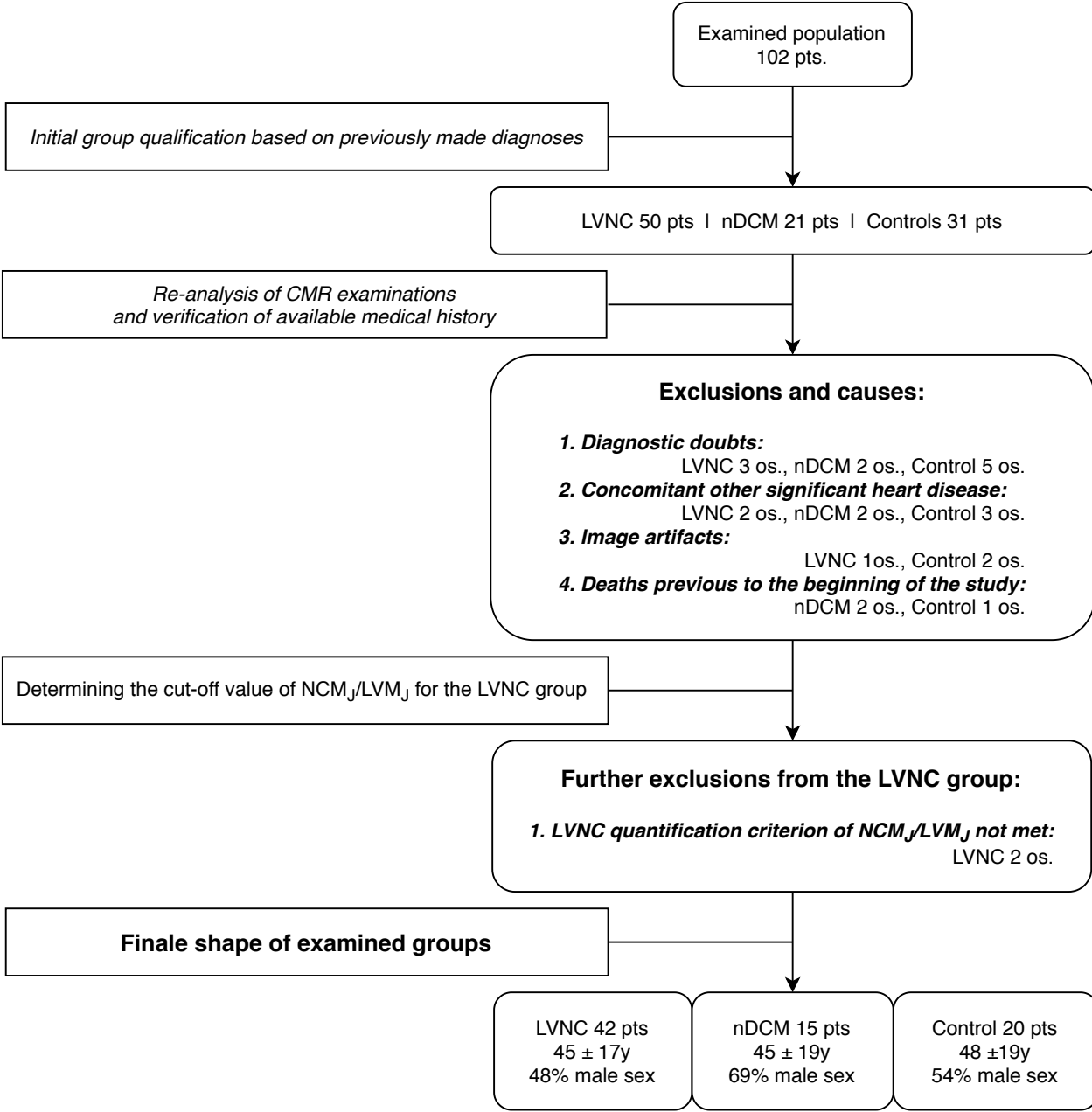

Supplement: S1 Fig — The stages of group qualification are marked with italics. LVNC–left ventricular noncompaction; nDCM–nonischemic dilated cardiomyopathy; Control–control group; pts.–patients. (PDF) [file pone.0230134.s001.pdf]
