# Supplementary material for: Influence of observer-dependency on left ventricular hypertrabeculation mass measurement and its relationship with left ventricular volume and ejection fraction – comparison between manual and semiautomatic CMR image analysis methods
Source: PLoS One. 2020 Mar 11;15(3):e0230134. doi: 10.1371/journal.pone.0230134 (PMC7065796; doi:10.1371/journal.pone.0230134)
Supplement: S1 Table — NCMH−noncompacted layer mass m. Hautvast’s computed algorithm [12]; NCMJ−noncompacted layer mass m. Jacquier et al. [10]; NCMJ/LVMJ−noncompacted/compacted layer mass ratio m. Jacquier et al. [10]; NCMH/LVMH−noncompacted/compacted layer mass ratio m. Hautvast’s computed algorithm [12]; EF–left ventricular ejection fraction; EDV–left ventricular end-diastolic volume. (DOCX) [file pone.0230134.s002.docx]

**Table A. Pearson’s correlation analysis between noncompaction mass measurements estimated by Hautvast’s and Jacquier’s approaches and left ventricular end-diastolic volume in the whole examined population and the LVNC group.**

|  | EDV (overall; N = 77) | | EDV (LVNC; n = 42) | |
| --- | --- | --- | --- | --- |
|  | r_Pearson’s_ | p | r_Pearson’s_ | p |
| NCM_H_ | 0.789 | <0.001 | 0.800 | <0.001 |
| NCM_H_/LVM_H_ | 0.434 | <0.001 | 0.391 | 0.010 |
| NCM_J_ | 0.799 | <0.001 | 0.816 | <0.001 |
| NCM_J_/LVM_J_ | 0.179 | 0.148 | 0.075 | 0.636 |

**Table B. Pearson’s correlation analysis between noncompaction mass measurements estimated by Hautvast’s and Jacquier’s approaches and left ventricular ejection fraction in the whole examined population and the LVNC group.**

|  | EF (overall; N = 77) | | EF (LVNC; n = 42) | |
| --- | --- | --- | --- | --- |
|  | r_Pearson’s_ | p | r_Pearson’s_ | p |
| NCM_H_ | -0.556 | <0.001 | -0.502 | <0.001 |
| NCM_H_/LVM_H_ | -0.349 | 0.004 | -0.153 | 0.334 |
| NCM_J_ | -0.572 | <0.001 | -0.491 | <0.001 |
| NCM_J_/LVM_J_ | -0.189 | 0.128 | 0.027 | 0.862 |
