# Supplementary material for: Influence of observer-dependency on left ventricular hypertrabeculation mass measurement and its relationship with left ventricular volume and ejection fraction – comparison between manual and semiautomatic CMR image analysis methods
Source: PLoS One. 2020 Mar 11;15(3):e0230134. doi: 10.1371/journal.pone.0230134 (PMC7065796; doi:10.1371/journal.pone.0230134)
Supplement: S2 Table — NCMH−noncompacted layer mass m. Hautvast’s computed algorithm [12]; NCMJ−noncompacted layer mass m. Jacquier et al. [10]; NCMJ/LVMJ−noncompacted/compacted layer mass ratio m. Jacquier et al. [10]; NCMH/LVMH−noncompacted/compacted layer mass ratio m. Hautvast’s computed algorithm [12]; EF–left ventricular ejection fraction; EDV–left ventricular end-diastolic volume. (DOCX) [file pone.0230134.s003.docx]

**Table A: Univariate regression analysis concerning left ventricular end-diastolic volume (EDV) for the overall examined group and the left ventricular noncompaction (LVNC) group.**

|  | EDV (overall, N=77) | | | | EDV (LVNC, n=42) | | | |
| --- | --- | --- | --- | --- | --- | --- | --- | --- |
|  | R^2^ | F | p_value_ | b | R^2^ | F | p_value_ | b |
| NCM_H_ | 0.622 | 105.39 | <0.001 | 0.789 | 0.640 | 71.25 | <0.001 | 0.800 |
| NCM_J_ | 0.338 | 112.62 | <0.001 | 0.799 | 0.667 | 79.98 | <0.001 | 0.816 |
| NCM_H_/LVM_H_ | 0.189 | 17.50 | <0.001 | 0.434 | 0.153 | 7.22 | 0.010 | 0.391 |
| NCM_J_/LVM_J_ | 0.032 | 2.139 | 0.148 | -0.180 | 0.053 | 3.619 | 0.062 | -0.230 |
| EF | 0.703 | 151.58 | <0.001 | -0.839 | 0.623 | 66.03 | <0.001 | -0.789 |

**Table B: Comparison of the multivariate analysis models concerning left ventricular end-diastolic volume (EDV) for the overall examined group and the left ventricular noncompaction (LVNC) group.**

|  | EDV (overall, N=77) | | | EDV (LVNC, n=42) | | |
| --- | --- | --- | --- | --- | --- | --- |
|  | R^2^ | F | p_value_ | R^2^ | F | p_value_ |
| EF + NCM_H_ | 0.825 | 148.41 | <0.001 | 0.808 | 81.89 | <0.001 |
| EF + NCM_J_ | 0.830 | 154.30 | <0.001 | 0.842 | 104.21 | <0.001 |
| EF + NCM_H_/LVM_H_ | 0.708 | 76.49 | <0.001 | 0.660 | 37.90 | <0.001 |
